# Replacement Beef Cow Valuation under Data Availability Constraints

**DOI:** 10.3389/fvets.2017.00185

**Published:** 2017-11-06

**Authors:** Amy D. Hagerman, Jada M. Thompson, Charlotte Ham, Kamina K. Johnson

**Affiliations:** ^1^United States Department of Agriculture, Animal and Plant Health Inspection Service, Veterinary Services, Fort Collins, CO, United States; ^2^Department of Agricultural and Resource Economics, University of Tennessee, Knoxville, TN, United States

**Keywords:** livestock valuation, data constraints, price forecasting, model comparison, bred cattle values

## Abstract

Economists are often tasked with estimating the benefits or costs associated with livestock production losses; however, lack of available data or absence of consistent reporting can reduce the accuracy of these valuations. This work looks at three potential estimation techniques for determining the value for replacement beef cows with varying types of market data to proxy constrained data availability and discusses the potential margin of error for each technique. Oklahoma bred replacement cows are valued using hedonic pricing based on Oklahoma bred cow data—a best case scenario—vector error correction modeling (VECM) based on national cow sales data and cost of production (COP) based on just a representative enterprise budget and very limited sales data. Each method was then used to perform a within-sample forecast of 2016 January to December, and forecasts are compared with the 2016 monthly observed market prices in Oklahoma using the mean absolute percent error (MAPE). Hedonic pricing methods tend to overvalue for within-sample forecasting but performed best, as measured by MAPE for high quality cows. The VECM tended to undervalue cows but performed best for younger animals. COP performed well, compared with the more data intensive methods. Examining each method individually across eight representative replacement beef female types, the VECM forecast resulted in a MAPE under 10% for 33% of forecasted months, followed by hedonic pricing at 24% of the forecasted months and COP at 14% of the forecasted months for average quality beef females. For high quality females, the hedonic pricing method worked best producing a MAPE under 10% in 36% of the forecasted months followed by the COP method at 21% of months and the VECM at 14% of the forecasted months. These results suggested that livestock valuation method selection was not one-size-fits-all and may need to vary based not only on the data available but also on the characteristics (e.g., quality or age) of the livestock being valued.

## Introduction

Livestock husbandry involves many production risks including disease, predators, and natural disasters. When such events occur on a large scale—as in the case of a large-scale natural disaster such as drought or blizzard or in the case of a multistate disease outbreak—the production losses have consequences beyond the farm gate, affecting local economies, associated industries such as processing, and consumers. Economists are often tasked with estimating the impacts associated with production shocks. Frequently, the first metric of impact estimated is the extent of death or reduced production and the subsequent financial losses to a livestock owner. These direct losses arise from death, abortion, or reduced productivity such as lower average daily gain or lower daily milk production. Ideally, valuation would be based on timely, comparable animal transaction data from an animal with similar type, age, and quality characteristics sold in the same regional cash market as the animal being valued. However, lack of available data or absence of consistent reporting reduces the efficacy of these valuations. Several factors limit the availability or usability of market data including market data accessibility, market reporting, market thinness,^1^ and integrated or closed-system farming. The extent to which data are constrained affects the options available for livestock valuation.

Factors associated with market data accessibility are the more obvious limitations of livestock valuation. The traditional livestock market model, a centralized location in which substantial numbers of buyers and sellers meet face-to-face to trade livestock, is still common in many parts of the world. Bids are public knowledge, and transaction data provide information for both public and private users (1). Market data accessibility poses a challenge to those livestock producers in isolated areas. In such situations, little transaction data are available for livestock in outlying towns and villages. When transaction data are available, high costs of getting animals to a central market location affect loss estimate accuracy, particularly on livestock that are raised for local consumption and were never destined for a centralized market. Even when auction markets are accessible, not all markets record and maintain transaction data on a regular basis. In volatile market conditions, the infrequent reporting of transaction data raises questions of the timeframe that is appropriate for estimating livestock values.

The increasing use of alternative marketing arrangements in lieu of traditional livestock markets further complicates the issue of livestock valuation. Transactions in livestock auction markets may still be seen as the primary price discovery mechanism; however, a trend of reduced utilization of markets threatens the usefulness of traditional market transaction data for pricing. For example, Joseph et al. (2) found that the futures market played the greatest role in price discovery in the United States fed cattle market from 2001 to 2012. Mathews et al. (1) similarly found that price discovery in the cattle market from 2008 to 2014 was largely driven by the cattle futures market in the United States, while cash transactions in traditional markets played a smaller, but still significant, role. Other market streams include virtual or online auctions, private treaty sales, and forward contracting.

Fully integrated, farm-to-table companies may not estimate the value of their intermediate stage livestock. Instead these companies focus on the value of the final retail product and the impact on their financial bottom line. In the United States, the cash market for poultry has virtually disappeared (1). The swine industry in the United States also exhibits a large degree of integration, and live swine are sold in a cash market less frequently. Consequently, concern exists for the accessibility of cash market swine prices for price discovery (3). Concerns over thinning cash markets have been discussed in the market literature for some time, and increasing consolidation in all sectors mean this discussion is likely to continue into the future.

Cash market values may inadequately capture the role of livestock as a personal capital asset (4), the impact livestock have in community supported agriculture, and the importance of livestock in a local food system. For example, a cow can provide milk, progeny and draft power in her lifetime plus meat and hide at the end of her productive life. Further the value of genetic stock, or seedstock, includes intangible characteristics such as investment in genetic improvement (5) or reputation-value (6). Valuing a cow’s loss with only her meat value at the market level will undervalue her role to a farm or community.

The complexities of livestock valuation increase when the disaster causing livestock losses has an impact on market prices. For example, for a highly contagious disease outbreak in a country with extensive exports, market prices can be driven down sharply. This situation reduces the usability of current market data to perform counterfactual analysis on disease price recovery. In such a case, it may be desirable to value livestock losses based on pre-disease prices or price forecasts based on pre-disease market information.

These factors affecting the availability or usability of market data reduces the accuracy of livestock production loss estimates, but it is difficult to quantify the extent of the inaccuracies. Certainly in some markets or for some animal types data are limited to such an extent that livestock loss values would be very difficult to estimate without primary data collection. Such a situation is not the focus of this article. In this study, we focused on quantifying the inaccuracies associated with limited data availability in livestock markets. To do this, we selected a market that currently has robust data. Then, we estimated values using alternative data and methods to mimic the impact of limited data availability. We seek to answer the questions: What if robust data were no longer available? What alternative methods could be used to estimate livestock values? How different are those estimates from the actuals?

Oklahoma bred replacement beef cows can easily be undervalued due to limited market reporting, market thinness, and closed-system farming. This article compared an observed Oklahoma bred beef cow price series to forecasted bred replacement beef cow values estimated with three alternative methods: hedonic pricing, vector error correction modeling (VECM), and cost of production (COP). Each method examined had benefits and drawbacks, especially when forecasting forward to determine future animal values.

## Background

### United States Cattle Production and Marketing

**Figure 1:**
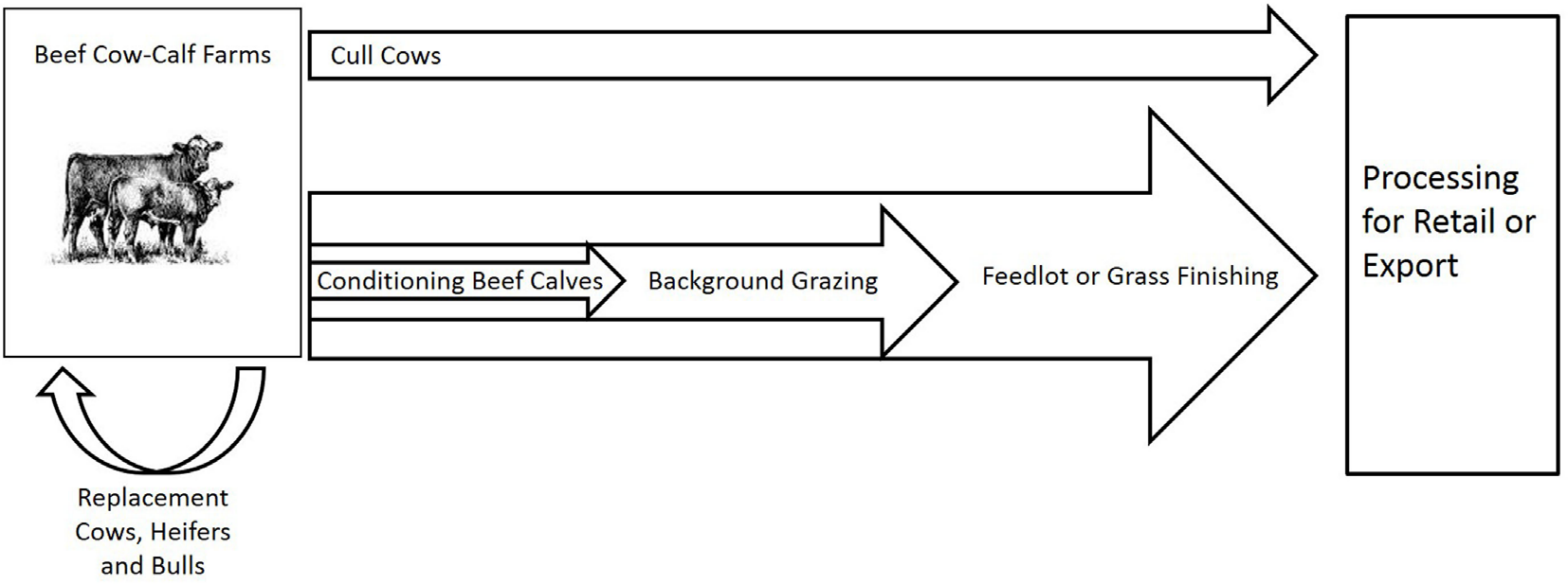
Conceptual flow of beef cattle production in the United States.

Beef cattle production in the United States is not as highly centralized as poultry and swine production. However, beef cattle production is the highest value livestock industry in the United States, generating $78.2 billion in cash receipts in 2015 when the United States cattle inventory was 89.1 million head (7). Cattle go through multiple stages of production and often change ownership multiple times as a consequence (Figure 1).

Beef cattle production begins on grass-raised cow–calf operations. In the 2012 United States Census of Agriculture, there were approximately 913,000 cattle operations in the United States with an average of 99.5 head of cattle and calves (7). Almost 82% of beef cow–calf operations in the United States were small, family owned enterprises with less than 50 head of cows. These small operations held almost 30% of beef cow inventory (7). Less than 10% of cows were held by large operations with more than 1,000 head; however, over 35% of cattle on feed were on large feedlots with more than 1,000 head (7).

Calves primarily sell to feeding operations (feedlots) before slaughter. At weaning, calves may go through a preconditioning or background grazing stage before dry lot feeding or calves may move directly to dry lot feeding. The remaining weaned calves retained for replacement—primarily heifer calves as well as a small portion of bull calves retained for breeding stock—either stay in the herd of birth or sold to other cattle operations. As of January 1, 2016, replacement beef heifers represented 32% of all heifers (7). Replacement cattle are sold via private treaty, production sales—public sales that are held by one or two seedstock producers—or through weekly or monthly public sale yard auctions. Spatial, quality, and time factors all affect United States beef cattle pricing. For example, Blank et al. (8) found that calf and yearling cattle prices were higher in Omaha, Nebraska relative to other parts of the Westerns United States with prices declining with each additional mile of distance from Omaha.

Oklahoma is both a location of extensive cow–calf production as well as an important geographical area for background grazing and cattle feeding for slaughter. Oklahoma collects and reports bred replacement cow and heifer data at seven markets reported through the United States Department of Agriculture Agricultural Marketing Service (USDA-AMS). Sales reports from these seven markets include detailed information on cattle sold as discussed in the data section.

### Market and Non-Market Methods

Market data on comparable animals would ideally be used for the valuation of replacement beef cow and heifer losses. Comparable, local transactions data would include characteristics for animal type such as age, weight, and quality. Most commonly, this type of data is available for cattle that are slaughter ready such as cows that have been culled due to reproductive issues and will be slaughtered for beef. These comparable animal transaction data are likely to be region specific and may possibly be privately held by an individual or company, making collection or extrapolation to other regions challenging.

Even when market data are available, forecasting prices forward is potentially necessary when (a) cattle losses in a single time period carry forward into future periods due to production effects (e.g., loss of a calf crop due to abortions will have an impact 2 or more years later) or (b) significant market price impacts may make the use of forecasted prices more appealing than the use of actual prices. Econometric approaches to forecasting allow the data to determine the structure of the model for valuation by taking advantage of long-term cause and effect relationships (9). When prices are known historically and are recorded in a detailed manner non-market valuation methods such as hedonic pricing can be used to estimate cattle values in future periods. Econometric methods, and vector error correction models in particular, assume that the same price dynamics will continue to apply into the future, which may not be true in large-scale disasters. While each of these methods has been used in isolation, none have been applied simultaneously to a replacement cattle data series to assess the implications of limited data availability on forecast accuracy. The methods examined here are summarized in Table 1.

**Table 1:** Alternative methods for estimating the value of livestock lost due to disasters or disease.

Valuation method	Description	Data needs	Benefits	Pitfalls
Observed market values of equivalent animal types	Value losses by using comparable sales at local, publically reporting livestock markets	Recent, local market reports with detail on animal type, age, and weight	Most closely estimates the value of lost livestock that could have been taken to the local market	Markets do not always sell comparable animal types. For extended or extensive natural disasters, market impacts of the event itself may change valuation and in the case of animal disease, will not represent the full losses

Hedonic pricing	Use of sales data to econometrically estimate the value of animal characteristics and attributes	Recent, local market reports with detail on animal type, age, breed, and other unique characteristics of the animal and market	Makes use of market reporting to reveal preferences for animal characteristics	If the animal of interest was not reported, this method cannot accurately predict value. If market data were not available a time intensive and costly survey must be used

Vector error correction model	Use of other livestock price series and input costs to estimate market values for thin or unobserved animal types in the market	Recent market reports on downstream animal products and inputs to production	Makes use of market data readily available and can be extrapolated to areas where data are less readily available	Still requires a price series at some geographic level as the dependent variable and makes the assumption that market structures are similar in the area being extrapolated

Cost of production (COP)	Value losses by calculating the sum costs incurred to raise an animal to a point in time and adds a proportion of proposed future profits back to the animal	Current annual total COP from enterprise budget, accompanying weaned calf price (if using purchased replacement heifer equation)	Data may be specific to a farm, local area or a point in time relative to more extensive time series data	Using basic enterprise budgets results in a single price point. Provides a floor price, not considering price fluctuations for inputs and outputs. Enterprise budgets are not available for all states/regions

#### Hedonic Pricing

Hedonic modeling is a well-established method for determining the intrinsic, revealed value of factors or characteristics contributing to heterogeneous market prices. Waugh (10) first presented a formalized model to link prices to product quality and characteristics, which was later incorporated into the consumer demand literature (11) and given theoretical foundations (12, 13). Hedonic modeling can use market data or can use values elicited from individuals through a survey. Using recorded prices for differentiated products or services, an estimate of the implicit value that observable and unobservable characteristics contribute to the total price of a good can be determined assuming these characteristics have underlying utility and the value of each characteristic contribute to the total cost of the good or service. In a competitive market, the final market price was determined through the market contract, where the price paid for a good or service was the tangential meeting of a buyers bid function and the sellers offer function. The general form may be written as follows:

(1) $$P_{it}=F(O_{it},U_{it})+\varepsilon_{it},$$

where *P* are the observed market prices, *O* are the observable characteristics, *U* are the unobservable characteristics, and ε was the disturbance term all for good *i* in time *t*. These characteristics can be any combination of quantitative and qualitative variables. Using econometric modeling, a formalized function can be estimated.

Hedonic pricing is an established method for valuing cattle based on their characteristics—primarily for feeder or fed cattle, though the literature for those cattle types will not be discussed here. The method has been applied less frequently in breeding cattle. For example, Parcell et al. (14) examined various cow attributes on cow–calf pair pricing in a hedonic modeling framework and found that various factors such as age, breed, size, and gestation status, among others, were significant in explaining pair value variation at auction in 1993. Recent studies that have examined breeding cattle have included an application of hedonic pricing on cow quality characteristics in Oklahoma bred cows by Mitchell and Peel (15) and Colorado breeding bulls by Kessler et al. (16). Mitchell and Peel in particular use the same data series used here to examine the marginal impact of quality and market location on bred cow prices. The authors did find significant, positive impacts on livestock value for younger, heavier, late gestation cows with higher quality.

Kessler et al. (16) looked explicitly at the impact of expected progeny differences (EPDs) and the ability to thrive in the high altitudes of the Colorado Rockies on breeding bull values. The authors found that values were higher for select performance measures such as high yearling weight and EPDs on weaning weight and milk production in progeny as well as a lower pulmonary arterial pressure score that indicated some lessened likelihood the bull would suffer from high altitude disease. Hedonic pricing has been used in other countries for livestock valuation of cattle. For example, Williams et al. (17) examined the impact of cattle characteristics on market values in West Africa and found that young breeding cattle had higher values than market cattle, and among market cattle young zebu steers with excellent body condition received the greatest market premium.

#### Vector Error Correction Models

If the sort of detailed transaction data used for hedonic pricing is not available, it is possible that more aggregate data may be available. In this instance, a multivariate time series method such as vector autoregressive modeling or VECM that uses economic theory to determine the interrelationships between known input prices and output prices can be used to forecast livestock valuations. Application of this approach for livestock valuation purposes does require that market data be available at a more aggregate level, but the price data do not have to be as detailed as required for hedonic pricing. The goal is to create the most accurate price forecast using the smallest reasonable list of variables that are economically significant (9) for that animal type. For livestock, explanatory variables would be expected to relate to upstream or downstream production, input costs, and consumption as well as longer term exogenous factors.

For example, in the United States, the National Agricultural Statistics Service collects the price received by producers for cows sold in a particular month in dollars per hundredweight. The same survey also collects the value of calves sold, as well as the pasture rental rate in dollars per acre. Lagged heifer calf prices and pasture provide in inputs to cow–calf production, and current calf prices and feeder calf prices serve as the values end products. However, each of these price series could be highly cointegrated and require special handling methodologically.

Multivariate regression analysis allows the interrelationships between commodities to determine value. The vector autoregressive model (VAR) in levels was introduced by Sims (18) and looks at dynamic response of variables to exogenous shocks that are important sources of economic fluctuations (19). Livestock price series often exhibit non-stationary error terms and may follow long-run interrelationships with other livestock price series. When such cointegration is present, first differences are used to achieve stationarity but an error correction term is included in the model that captures the long-run equilibrium position directly. When an error correction term is added to a VAR, the resulting model is a vector error correction model (VECM) as first suggested by Engle and Granger (20). It should be noted that Phillips and Durlauf (21) argued if data are both non-stationary and cointegrated differencing is not necessary, meaning a VAR could be used. Based on the Johansen’s cointegrated vector autoregressive model with *k* lags (22), the data generating process of *Y_t_* that is a *n*-by-1 vector of price series in time *t*, can be modeled as a VECM with lags from *i* to *k* − 1:

(2) $$\Delta Y_{t}=\Pi Y_{t-1}+\sum_{i=1}^{k-1}\Gamma_{i}\Delta Y_{t-i}+\sum_{l=1}^{J}\theta_{l}D_{l}+\;\varepsilon_{t},$$

where Δ*Y_t_* was a *n*-by-1 vector of first-order difference of prices, *Y_t_*_−_*_i_* was the vector of lagged own commodity prices, Π was the *n*-by-*n* cointegration rank matrix, Γ was a *n*-by-*n* matrix of parameters on the lagged price differences, *D_l_* was a matrix of dummy variables to represent seasonal or cyclic trends that has a value of 1 in period *l* and 0 otherwise, and ε was a *n*-by-1 vector of error terms in time *t* (23). Detailed descriptions of VECM models can be found in Kennedy (19) and Lütkepohl (23).

As consolidation in livestock industries increased in the 1980s, analyses of market cointegration became more common. In cattle markets, cointegration has been identified between cash and futures live cattle prices (24), among regional slaughter cattle markets (25), and the impact of mandatory price reporting on regional market cointegration (26). VAR and VECM models have been used to forecast livestock prices for feeder and fed cattle, particularly when simultaneously estimating multiple, cointegrated price series. For example, Fanchon and Wendel (27) looked at the ability of VAR, VECM and Bayesian parameter estimates to forecast feeder cattle prices in Kansas City (400 lb steers and 600 lb steers) and Omaha (1,000 lb steers) as well as the Omaha corn price and a monthly time trend. Forecast quality was tested using mean squared error (MSE), and results indicate that the VAR had the smallest MSE across 4 years but the VECM performed better in the long-run. Although VECM has been applied successfully at the national level, little quantitative information is available on the degree to which national data will over or underestimate a price forecast applied regionally.

#### COP Method

Application of time-series econometric approaches such as VECM is possible when frequent transaction data are available, but sometimes the available market data are insufficient. In such a situation, another option is to consider producers’ costs of production. This non-market valuation method estimates unobserved market values for livestock sold using the total expenses incurred to produce an animal plus a profit margin. Enterprise budgets provide annual estimates of income and expenses for specific production types and species and are usually updated annually for producers to use as an interactive planning tool for the following year. All price variation is captured during these annual updates. Estimates are calculated on an annual basis and represent the average income and expenses for a representative operation for that geographical area (usually state). Expenses are broken into variable and fixed costs, then the sum of these costs was used to proxy a break-even price for a cow. The challenge was determining the net profit margin for breeding livestock, which differs depending of the life stage of the breeding cow. The COP value estimate equation can be written as follows:

(3) $${\text{COP}}_{it}=C_{it}+\pi_{it},$$

where COP was the estimated value for a cow using the COP approach, *C* was a vector of costs incurred to maintain the cow as she raises a calf *i* in each time period *t*, and π is the profit earned for the calf produced *i* in each time period *t* plus her cull value at the end of her productive life.

Due to simplifying assumptions using the enterprise budgets there are some limitations of the COP approach when comparing that model to reality based on input and sales transactions in the daily marketplace. Enterprise budgets were calculated on an annual basis such that prices of inputs and outputs were fixed and did not fluctuate throughout the year. In addition, enterprise budgets were built to illustrate a representative operation based on the average costs and returns for producers in the area. Because of the longer production cycle, enterprise budgets for cattle do not capture all costs incurred and income generated in the same year for the same animal. So, there is a delay from when prices are realized at sale and when expenses are incurred to produce the animal being sold at a given point in time. In reality profit margins fluctuate across producers and across time while the COP approach uses the simplifying assumption that the annual average profit earned in a given year was the same earned in previous years.

## Materials and Methods

The availability or usability of market data affects the accuracy of loss estimates, but it is difficult to say the extent of the inaccuracies. We examined comparable animal transaction data, bred replacement beef cows in Oklahoma and estimated bred replacement beef cow values under alternative methods of hedonic pricing, VECM, COP, and nearest proxy of slaughter cow data. It was recognized that these methods still required larger amounts of data than may be available in some areas; however, each method did have different data intensity and used different types of data. Even where very little data are available, the comparison of these methods provides the parameters for animal valuation that could potentially be collected through primary data collection.

### Observed Price Series Data

The actual data used to measure the accuracy of each method evaluated here were a weekly bred replacement beef cow price series from Oklahoma auction markets. The bred replacement beef cow values are for seven traditional auction markets in Oklahoma reported by the Agricultural Marketing Service: Ada report KO_LS757 (28), Apache report KO_LS754 (29), El Reno report KO_LS751 (30), Oklahoma City report KO_LS750 (31), McAlister report KO_LS752 (32), Tulsa report KO_LS760 (33), and Woodward report KO_LS753 (34). The state of Oklahoma was split into East and West regions. East was Ada, Tulsa and McAlister. West was Apache, El Reno, Oklahoma City and Woodward. Each of these auctions sell bred replacement cows one day per week. The weekly transaction data from 2000 to 2015 were collated by the Livestock Market Information Center, and that data series was amended with data for 2016 from AMS report number KO_LS794 (35) which summarizes data from all seven markets.

At these auctions, cows are sold in lots, or groups, of cows that share similar quality or characteristics which were subjectively assigned based on attributes including conformity of size, weight, and visual inspection of cows at the market. This increases the likelihood the seller will receive a higher overall price for the lot of cows as buyers generally prefer to buy a homogeneous group of cows. In these types of auctions where commercial cows are sold, it was rare for cows to be sold individually. Individual sales of cows are more common in private liquidation sales or sales of seedstock cows—cows that will be used to produce other replacement breeding cattle. The factors included in the models included averages for each lot of cows for age, weight, calf weight, and gestation months where applicable. Indicator variables were put in for high quality cows (quality.high), cows that are above average but not quite high quality (quality.highaverage), cows that were below average but not quite low quality (quality.averagelow), and cows that were low quality (quality.low). Cows that were average quality were incorporated in the constant term. HideColor was specifically related to premiums associated with Angus breed-influence cattle in the United States and the popularity of Certified Angus Beef. Black was the predominant hide color due to the popularity of Angus cattle in commercial beef production, so hide color was specified as either “black” or “not-black” by the auction. Other indicator variables for the type of lot–cow only (lot.cow), heifer only (lot.heifer), and cow–calf pairs in the constant—as well as an indicator for Western Oklahoma markets (West) were defined. Indicator variables were also developed for the fixed effects for year, month, and global recession. Since cows are sold in lots, the transaction data describe price ranges (*P*_OK_) for lots of cow with similar characteristics (Table 2).

**Table 2:** Data description from the Oklahoma bred replacement beef cow data from 2000 to 2016 (20,602 observations).

Name	Notation	Description	Unit	Mean	Range
**Dependent variable**					
Price	*P*_OK_	The price per head for cows in Oklahoma	US dollars per cow	$1,049	$285–$3,800
**Explanatory variables**					
Region of Oklahoma	West	Binary variable to indicate the West region of Oklahoma	0.1	0.68	0–1
Age range	Age	The cow age for a given set of lots sold in an auction day	Years	5.5	1–12
Weight range	Weight	The cow weight for a given set of lots sold in an auction day	Pounds	1,114	570–1,850
Calf weight range	Calf.Weight	The calf weight for a given set of lots sold in an auction day	Pounds	186	50–1,125
Gestation range	Gestation	The gestation for a given set of lots sold in an auction day	Months	5.4	1–9.5
Quality characterization: high	Quality.High	Binary variable to indicate that the quality of the cows in a given lot was high	0.1	0.21	0–1
Quality characterization: high-average	Quality.HighAverage	Binary variable to indicate that the quality of the cows in a given lot was high-average	0.1	0.07	0–1
Quality characterization: average-low	Quality.AverageLow	Binary variable to indicate that the quality of the cows in a given lot was average-low	0.1	0.01	0–1
Quality characterization: low	Quality.Low	Binary variable to indicate that the quality of the cows in a given lot was low	0.1	0.02	0–1
Hide color	Hide.Color	Binary variable to indicate that cows in the lot had black hide	0.1	0.41	0–1
Lot type: cows	Lot.Cows	Binary variable to indicate that the type of the cows in a given lot are bred or open cows	0.1	0.65	0–1
Lot type: heifers	Lot.Heifers	Binary variable to indicate that the type of the cows in a given lot is heifers	0.1	0.06	0–1
Lot type: pairs	Lot.Pairs	Binary variable to indicate that the type of the cows in a given lot is cow–calf pairs	0.1	0.29	0–1

The observed data from Oklahoma provide an important resource for valuing beef cattle losses in that state. However, if that data source were not available it is possible to value Oklahoma beef cows based on other data sets. Each year the United States Department of Agriculture’s National Agricultural Statistics Service (USDA-NASS) surveys producers on prices received and expenses paid for various types of operations. USDA-NASS data from the same survey are available for cow sales in Oklahoma, but to proxy the effect of only having a national average value the United States average price received was analyzed.

**Figure 2:**
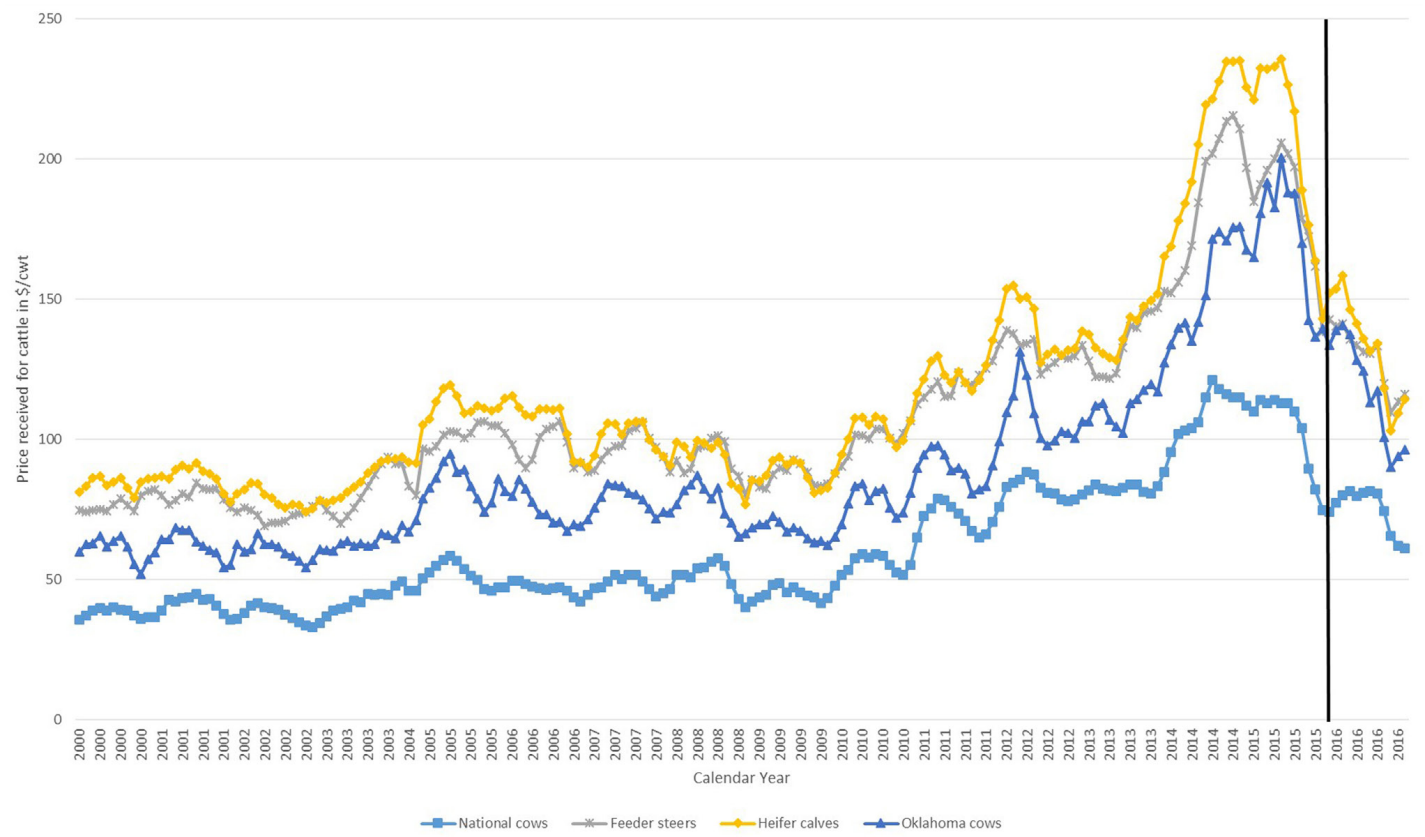
Price received ($/cwt) for cattle by calendar month from 2000 to 2016. Prices received nationally for cows on average in a single month (national cows), national prices received for feeder steers on average in a single month (feeder steer), national prices received for heifer calves on average in a single month (heifer calves), and Oklahoma prices received for bred cows in a single month (Oklahoma cows). The vertical black line indicates the observed prices used for the within-sample forecast comparison. Source: USDA NASS for all except Oklahoma cows. USDA-AMS for Oklahoma cows.

This national data would represent mild data constraints for valuing bred replacement cows in Oklahoma—a situation in which an analyst might believe that reasonably accurate livestock values could still be achieved. Namely, the national USDA-NASS data suffer from a lack of regionally specific data (data accessibility), a high degree of aggregation since bred replacement cows are combined with all other cow sales (market thinness), and a lack of quality characteristics (capital asset value). However, the data should have appropriately captured the underlying market fundamentals that move prices, as illustrated in Figure 2. There are regions of the United States that have higher prices for replacement beef cows than others, namely the northern and central plains states. Aggregation across the United States results in data that are expected to undervalue cows from Oklahoma. Further these data were not specific to bred replacement beef cows, but rather averaged across all cows sold in a particular month. Since the majority of cows sold from an operation are older cows that no longer having value as breeding animals, and will consequently be slaughtered, the resulting average price was expected to undervalue bred replacement beef cows. As discussed in section 2.2.2, the VECM estimates value based on upstreamand downstream prices. Table 3 provides summary statistics on these data.

**Table 3:** Data description from the national average sales price for cattle and national average pasture rental rate from 2000 to 2016 (204 observations).

Name	Notation	Description	Unit	Mean	Range
**Cointegrated dependent variables**					
National cow price	*P*_US_	The monthly price per hundredweight for cows in the United States	US dollars per hundredweight	$61	$33–$121
National heifer calf price	Hcalf	The monthly price per hundredweight for heifer calves in the United States	US dollars per hundredweight	$117	$74–$235
National feeder steer price	Fsteer	The monthly price per hundredweight for feeder steer^a^ calves in the United States	US dollars per hundredweight	$110	$69–$215
National pasture rental rate	Rent	The annual price per acre for renting pasture land for grazing	US dollars per acre	$11	$8.5–$14

**Explanatory variables**					
Fourth quarter	Qtr4	Binary variable that indicates October, November or December	0.1	0.25	0–1
Drought	Dro	Binary variable that indicates years in which pasture conditions were strained due to extraordinary drought	0.1	0.06	0–1
Great recession	GR	Binary variable that indicates the years of the Great Recession (December 2007–June 2009) when financial conditions for leveraging the purchase of cattle were poor	0.1	0.09	0–1

*^a^Feeder steer calves are castrated male cattle that have already been weaned and will enter conditioning, background grazing, and/or dry lot feeding for eventual slaughter*.

The USDA-NASS survey data were used to obtain the price cows sold from operations (PUS), for outputs of cow–calf production, and the inputs of cow–calf production. Prices for weaning weight beef heifer calves—500–600 lb feeder heifer calves (Hcalf)—and feeder steers—500–600 lb feeder steer calves (Fsteer)—were used as the downstream product of cow–calf ranching. Franchon and Wendel (36) used a corn feed price for input cost. While this may be appropriate at certain times of the year when supplemental feeding occurs, pasture is the primary feed source for cows in the United States. The inputs of cow–calf production for the purposes of this study were replacement heifers (lagged Hcalf) and pasture rental rates (Rent).

In addition to a time trend, an indicator variable for the fourth quarter of the year (Qtr4) was included. The majority of cows in the United States calve in the spring months. Calves are weaned in the fall, and culling decisions are often made immediately following weaning so that cows are not fed through the winter months. Since the United States cow data include the sale of cull cows, the fourth quarter of the year was controlled for. Exogenous factors affecting cow–calf operations were also considered as explanatory variables. Drought (Dro) results in stressed pasture conditions, which can lead producers to reduce the size of their cow herd. This in turn has an impact on the price of cows in the national dataset. Finally, the great recession (GR) that started in December 2007 and is generally considered to have ended in June 2009 had an impact on financial institutions. Many cow–calf producers in the United States depend on borrowing to buy cows. In the wake of the GR, that borrowing may have been more difficult to obtain.

### Models

The models will be applied and compared using four profiles of cattle (Table 4). These cattle are all black hide, in the West region of Oklahoma (see Table 2), and average-low, average or average-high quality.

**Table 4:** Specific equations for cattle types.

Cattle type	Cost of production (COP) equation—purchased heifer	COP equation—retained heifer
Weaned calf to 1-year-old	Cost to purchase weaned heifer calf + total cost to maintain calf for 5 months	Total cost to maintain heifer for 12 months + total cost to maintain calf for 5 months
2-year-old Replacement heifer	Weaned calf cost + total cost to maintain heifer for 12 months	Weaned calf cost + total cost to maintain heifer for 12 months
5-year-old Brood cow	2-year-old replacement heifer cost + total cost to maintain cow for 36 months − revenue from 3 calves	2-year-old replacement heifer cost + total cost to maintain cow for 36 months − revenue from 3 calves
10-year-old Cull cow	5-year-old Brood cow cost + total cost to maintain cow for 60 months − revenue from 5 calves	5-year-old Brood cow cost + total cost to maintain cow for 60 months − revenue from 5 calves

#### Hedonic Pricing

In terms of production, knowing the market price and the factors that contribute to the market price can help give an indication of the expected value of an animal with certain characteristics. For this work, factors from recorded market data provide an indication of the expected market value for cattle not traded on the market. The factors included are outlined in Table 2 of the data section as well as time fixed effects that account for potential temporal effects on market prices. The implicit contribution each variable makes to the price of cattle sold on the cash market was estimated. The empirical model estimated was written as follows:

(4) $$\begin{array}{c} P_{\text{OK}}=\beta_{0}+\beta_{1}\text{Age}+\beta_{2}\text{Weight}+\beta_{3}\text{CalfWeight}+\beta_{4}\text{Gestation}\\ \;+\beta_{5}\text{Quality.High}+\beta_{6}\text{Quality.HighAverage}\\ \;+\;\beta_{\text{7}}\text{Quality.AverageLow}+\beta_{8}\text{Quality.Low}+\beta_{9}\text{HideColor}\\ \;+\;\beta_{10}\text{Lot.Cow}+\beta_{11}\text{Lot.Heifer}+\beta_{12}\text{West}+\gamma T+\varepsilon , \end{array}$$

where *P*_OK_ were the average observed Oklahoma market prices per cow variables as defined in Table 2, *T* was a matrix of time fixed effects for year, month, and the global recession, β and γ were respective coefficients, and ε was the disturbance term. The base animal included in the constant term was a cow from a paired lot of average quality cows. The use of time fixed effects captured any structural breaks in the data as well as seasonal trends. This model was estimated using pooled ordinary least squares to capture the variation around the full weekly data as opposed to a panel data approach which would have required aggregation or averaging across cow lots per market before estimation due to the multidimensionality of the data.

#### Vector Error Correction Model

The products of cow–calf ranching are calves for consumption or replacement and cull cows. Prices of inputs and outputs of cow–calf production were included as described in the data section and Table 3. Output prices and input prices were included since the production function of outputs—in this case cattle sold—were influenced by the physical inputs and factors of production or in this case by feed prices and the prices of replacement cattle. The data were first tested for structural breaks using the Zivot–Andrews test (37), and a structural break was identified in November 2005. It is likely that this structural break indicates the point when United States markets began to recover from the December 2003 cow identified with typical bovine spongiform encephalopathy and prices for cows in the United States began to improve. The focus of this article was on forecasting into 2016, so the data were truncated at the structural break and the VECM was applied to the remaining 121 monthly data point between November 2005 and December 2015.

Several tests needed to be performed to specify the appropriate number of lags and rank of the VECM—augmented Dickey–Fuller (ADF) and Phillips–Perron (PP) unit root tests; Akaike information criteria (AIC), Schwarz Bayesian information criteria (SBIC), and Hannan–Quinn information criteria (HQIC) lag tests; and Johansen’s maximum likelihood method for cointegration. Greater detail on these tests can be found in Kennedy (19). The ADF and PP unit root tests examined the null hypothesis of a unit root against the alternative of a constant deterministic trend. Results from both tests indicated the presence of unit roots in all of the price data series in levels. However, when the same tests were run on first differences, the null hypothesis was rejected at the 1% confidence level. Thus all of the price series contained unit roots in levels but first-order differences were stationary, and thereby the variables in the series are I(1).

A VAR was specified and examined for 1, 2, 3, and 4 lags in each variable. The AIC, SBIC, and HQIC were used to determine the optimal number of lags by finding the lag that minimizes the AIC, SBIC, and HQIC. The optimal number of lags was 3 under each of the three criteria. Johansen’s maximum likelihood method for cointegration was used to determine the optimal rank of the error correction term for the VECM. The rank determines the order of cointegrating vectors included in the VECM. Johansen’s cointegration test indicated the presence of cointegration and that a rank of 2 be used in the VECM form specified in Eq. 2.

#### Cost of Production

Since this comparison was using the Oklahoma price series data, we provided an example of the application of the COP method using an enterprise budget developed by Oklahoma State University (38) for a typical Oklahoma cow–calf ranching operation in 2016. This budget represented the expected income and expenses for an average herd in this area and was updated annually using historical data and specialist recommendations, but could also be modified based on user specific parameters. Variable costs included feed, supplements, veterinary supplies and services, marketing, machinery, labor, and other. Fixed costs included machinery, value of breeding stock, and land. The sum of these categories gave the estimate for the total annual costs at $714.73 per head. For this analysis, we assumed that this cost was held constant across the years explored (i.e., no change in input prices) to understand how values using the COP method change over the duration of a cow’s life.

Another assumption was that each cow has a live calf that was weaned and then sold at market (i.e., no death loss). The annual expenses of feeding, getting pregnant and birthing, and keeping the cow healthy were offset by the revenue generated from selling the calves at market each year resulting in profit. For this analysis, we also assumed a fixed price received from weaned calves of $880 (i.e. no change in output price), so the annual profit for years 3 through 10 is $165. Essentially this profit was removed from the value of the cow each year when using the COP valuation method.

Specific equations are presented in Table 4 for each cattle type. The first year started with valuing a weaned calf. For purchased replacements this was the cost of the weaned calf plus 5 months of costs to maintain that calf assuming they were weaned at 210 days [$714.73*(5/12) = $297.80]. Each following year an additional $714.73 was added in cost and the assumed revenue generated from the sale of her calf, $880, was subtracted for the years she will produce a calf (replacement heifers usually do not produce a calf until their third year). The revenue generated from the calves sold represented the amount the market was willing to pay for that calf, which included the profit margin that was received for raising that calf to weaning. A cow was considered an asset that was producing revenue each year through the sale of calves. This income covered the annual cost of producing the calf, the annual cost of maintaining the cow, and some of the initial investment cost, or capitalization cost, of raising a heifer to maturity as a replacement female in that herd. These values were aggregated to create a cumulative value over time for this cow until she was 10 years old.

## Results

### Hedonic Pricing Method Results

Select results for the hedonic pricing method are presented in Table 5. Time fixed effects were not presented, but can be provided by the authors. Results were presented in absolute terms, such that the value was interpreted as the dollar change in the average market price of a cow sold in a lot in response to a change in the respective variable. For example, the market price of a cow was estimated to increase by $0.58 per pound.

**Table 5:** Hedonic estimation of replacement beef cow prices in Oklahoma (2002–2015).

Variable^a,b,c^	
Age	−22.93*** (1.04)
Weight	0.58*** (0.02)
Calf.Weight	0.98*** (0.09)
Gestation	2.81* (1.64)
Quality.High	189.91*** (5.94)
Quality.HighAverage	148.64*** (8.95)
Quality.AverageLow	−30.15 (18.99)
Quality.Low	−99.17*** (11.07)
HideColor	37.07*** (4.17)
Lot.Cows	−94.60*** (18.69)
Lot.Heifers	−60.42*** (20.60)
West	8.68*** (4.32)
Constant	−742,537.49*** (154,929.87)
Observations	22,187
*R*-squared	0.875

*^a^Time effects are not presented, but can be requested from authors*.

^b^Robust SEs in parentheses; ***p < 0.01, **p < 0.05, *p < 0.1.

*^c^Detailed descriptions of these variables are found in Table 2*.

As expected, results showed there were preferences for age, weight, quality, breed type, and lot type. The extent and direction of these preferences vary by factor, but all significantly affected the market price. Age negatively affected market price by $22.93 per cow as the average age of a cow in a lot increases by a year, implying buyers accounted for future calving potential when purchasing cows. Inversely, average weight and calf weight positively affected market price by $0.58 and $0.98 per pound respectively. This implies a preference for large bodied cows and larger calves. Preference for larger calves was two-fold. First, larger calves typically coincided with larger bodied breeds. Second, weight was proportional to age, wherein larger calves tended to be older and thus a shorter period before the calf represented earning potential through breeding or slaughter.

In line with expectations, gestation months had a positive effect on market price, $2.81 per month of gestation. At most, this was an increase in the price of a cow by $26.70. This perhaps indicated a slight preference for a bred cow, which represented additional earning potential in the form of the calf being carried. This value did not reflect the earning potential of a short-bred cow sold with an unweaned calf at side since most of the reports did not list the cow’s stage of gestation.

Binary indicator variables helped identify preference for traits of cows being sold. Determined quality of cows being sold showed a premium for high quality ($189.91) and high-average ($148.64) over average quality. As expected, average-low to low quality had a lower price than cows of average quality. Black hide color also increased the value of a cow in a given lot by $37.07. Location had a positive impact on price. Cows sold in western Oklahoma tended to be $8.68 more than cows sold at auctions in eastern Oklahoma, accounting for all the other variation.

In terms of preferences for the type of lot sold, pairs appeared to be the most preferential. Lots of only cows, bred and open, were expected to decrease the value of a cow by $94.60 than a lot of pairs reflecting the added value of the calf at side. Heifers had a less steep difference of $60.42 per head. This preference may have been driven by potential earnings. Each attribute contributed some intrinsic value to the price of a cow. Using the hedonic pricing method, these values were revealed. For price forecasting, the attributes could be combined and a value could be approximated for various replacement beef cow and heifer types.

### Vector Error Correction Model Results

Short-run results from the VECM are presented in Table 6 for cows and Table 7 for heifers. The cointegrating equations were less than 1 and significant for the first-order differences of both cows and heifer calves, which was to be expected given the results of Johansson’s cointegration test. The short-run effect was based on regression of the first-order difference in the price of cows against lagged prices of explanatory variables as shown in Eq. 2. The AIC, SIC, and HQIC recommended three lags in the model, thus the regression on the first-order differenced dependent variable will include 2 (or *k* − 1) lags. The explanatory variables explained 64% of the variation for cows in the short-run as measured by the *R*^2^. Cow price responded to feeder steer price, pasture rental rate, and the fourth quarter indicator. As feeder steer price increased, cow price declined a month later. This may have indicated the slight lag in the seasonal market cycles of cows as compared with feeder cattle. As pasture rental rate per acre increased, cow price increased two months later. This was a reasonable relationship since increased rental rates could have served as an indicator of higher demand for calves. In the fourth quarter of the year, cow prices declined. It was expected that more cows are sold in the fourth quarter after the majority of calves are weaned in the fall, resulting in a decline in prices.

**Table 6:** Vector Error correction model short-run effects in Δ*P*_US_.

Variable^a,b^	Coefficient (SE)	*t*-Statistic
Cointegrating equation 1	0.10 (0.034)	2.96***
Cointegrating equation 2	0.09 (0.05)	1.88*
Constant	0.28 (0.21)	0.13
*P*_US,_*_t_*_−1_	0.10 (0.13)	0.80
*P*_US,_*_t_*_−2_	−0.03 (0.10)	−0.24
Hcalf*_t_*_−1_	0.076 (0.10)	0.75
Hcalf*_t_*_−2_	0.074 (0.10)	0.72
Fsteer*_t_*_−1_	0.21 (0.11)	1.85*
Fsteer*_t_*_−2_	−0.002 (0.12)	−0.02
Rent*_t_*_−1_	−0.33 (1.05)	−0.31
Rent*_t_*_−2_	2.25 (1.08)	2.10**
Qtr4*_t_*_−1_	−3.86 (0.88)	−4.40***
Qtr4*_t_*_−2_	−1.19 (0.77)	−1.55
Dro*_t_*_−1_	0.68 (1.61)	0.42
Dro*_t_*_−2_	2.00 (1.58)	1.26
GR*_t_*_−1_	0.17 (1.63)	0.10
GR*_t_*_−2_	1.31 (1.62)	0.81

*^a^“*P*_US_” is the monthly price per hundredweight for cows in the United States. “HFeeder” is the monthly price per hundredweight for heifer calves in the United States. “SFeeder” is the monthly price per hundredweight for feeder steer calves in the United States. “Rent” is the annual price per acre for renting pasture land for grazing. “Qtr4” is a binary variable that indicates October, November, or December. “Dro” is a binary variable that indicates years in which pasture conditions were strained due to extraordinary drought. “GR” is a binary variable that indicates the years of the Great Recession (December 2007–June 2009) when financial conditions for leveraging the purchase of cattle were poor. More details are provided in Table 3*.

*^b^The subscript “t − 1” indicates a 1 month lag in prices or a binary variable value from the previous month. The subscript “t − 2” indicates a 2-month lag in price or a binary variable value from 2 months previous*.

**Table 7:** Vector error correction model short-run effects in ΔHCALF.

Variable^a,b^	Coefficient (SE)	*t*-Statistic
Cointegrating equation 1	0.24 (0.09)	2.79***
Cointegrating equation 2	0.24 (0.12)	1.98**
Constant	−0.01 (0.08)	−0.03
*P*_US,_*_t_*_−1_	−0.34 (0.32)	−1.05
*P*_US,_*_t_*_−2_	−0.16 (0.26)	0.61
Hcalf*_t_*_−1_	0.09 (0.26)	0.33
Hcalf*_t_*_−2_	0.14 (0.26)	0.54
Fsteer*_t_*_−1_	0.45 (0.28)	1.59
Fsteer*_t_*_−2_	0.02 (0.29)	0.08
Rent*_t_*_−1_	−1.53 (2.65)	−0.58
Rent*_t_*_−2_	2.76 (2.72)	1.01
Qtr4*_t_*_−1_	−6.06 (2.22)	−2.73***
Qtr4*_t_*_−2_	−3.91 (1.94)	−2.02**
Dro*_t_*_−1_	−1.52 (4.08)	−0.37
Dro*_t_*_−2_	2.05 (4.01)	0.51
GR*_t_*_−1_	−4.69 (4.11)	−1.14
GR*_t_*_−2_	4.98 (4.10)	1.21

*^a^“*P*_US_” is the monthly price per hundredweight for cows in the United States. “HFeeder” is the monthly price per hundredweight for heifer calves in the United States. “SFeeder” is the monthly price per hundredweight for feeder steer calves in the United States. “Rent” is the annual price per acre for renting pasture land for grazing. “Qtr4” is a binary variable that indicates October, November, or December. “Dro” is a binary variable that indicates years in which pasture conditions were strained due to extraordinary drought. “GR” is a binary variable that indicates the years of the Great Recession (December 2007–June 2009) when financial conditions for leveraging the purchase of cattle were poor. More details are provided in Table 3*.

*^b^The subscript “t − 1” indicates a 1 month lag in prices or a binary variable value from the previous month. The subscript “t − 2” indicates a 2-month lag in price or a binary variable value from 2 months previous*.

Explanatory variables did a poorer job explaining variability in heifer calf prices with an *R*^2^ of 40%. This may be the result of input and output variables being customized to cow–calf production. It was also likely that other variables, such as the price of beef, were affecting heifer calf prices. The only variable that significantly explained the variation in the first-order differenced heifer calf price was the indicator for the fourth quarter. The majority of calves are weaned in the fall, gaining weight—and value—throughout the following winter, spring and summer.

The coefficients of the cointegrating equations in Tables 6 and 7 indicated that the short-run relationships did adjust back to the long-run equilibrium. The speed of that adjustment was indicated by the coefficients in Table 8. In absolute values, smaller adjustment coefficients indicated a faster movement back to a stable long-run equilibrium. Expectations based on output prices, such as feeder steer prices, were adjusted to more quickly than exogenous shocks, such as drought. The fourth quarter, drought, and rent adjustment coefficients indicated a slower move back to equilibrium. Only the adjustment coefficient for the GR was insignificant in the long-run.

**Table 8:** Vector error correction model long-run effects.

Variable	Coefficient (SE)	*z*-Statistic	Coefficient (SE)	*z*-Statistic
Constant	64.07		−24.36	
*P*_US_	1		0	
Hcalf	8.9 e−16		1	
Fsteer	−0.46 (0.11)	−4.33***	−1.30 (0.08)	−15.49***
Rent	−8.75 (3.74)	−2.34**	5.84 (2.99)	1.96*
Qtr4	92.92 (7.34)	12.66***	−52.10 (5.86)	−8.88***
Dro	−15.74 (6.13)	−2.57***	6.07 (4.90)	1.24
GR	−2.31 (5.62)	−0.41	4.32 (4.49)	0.96
Chi-squared	338.6918***		896.79***	

***p < 0.01, **p < 0.05, *p < 0.1.

To forecast, results on the short-run coefficients were forecast forward based on the monthly cow and calf price series in the short-run but accounted for long-run adjustment to feeder steer price, rent, the presence of drought and the fourth quarter to attain long-run stability.

### COP Approach Results

The COP approach estimated an annual value for these animals and results are presented in Table 9. This approach revealed the break-even point when enough calves have been sold to pay for the costs that have been incurred to raise a female to sexual maturity, through gestation, and up to weaning her first calf. This was important since no income was received for a female up weaning her first calf.

**Table 9:** Annual cost-of-production results for 2016.

Cattle type	Purchased heifer	Retained heifer
Weaned calf to 1-year-old	$1,178 (184%)	$1,013 (158%)
2-year-old Replacement heifer	$1,893 (187%)	$1,727 (170%)
5-year-old Brood cow	$1,397 (120%)	$1,231 (106%)
10-year-old Brood cow	$570 (62%)	$405 (44%)

The cost for purchased heifers was always higher than retained heifers; however, the market only records the price for heifers sold from operations. Prices for replacement heifers sold at market incorporates the split of future earning potential between the buyer and the seller, allowing the seller to realize some profit from heifer sales. However, purchase of heifers incurs cost as well. Since the value of retained heifers was based on the cost incurred to produce that heifer, it makes sense that the COP approach would value purchased heifers higher than retained heifers reflecting the additional cost of purchase. If the portion of earning potential was accounted for as well, the $166 difference between purchased and retained 2-year-old replacement heifers would not occur in theory.

The market prices for cull animals were driven by beef demand and supply. The 10-year-old brood cow represents a fully depreciated cow in the COP method and may not reflect her remaining earning potential. This COP approach only took into account the maintenance cost, breeding cost, and the realized profit from selling her calves each year. Since the use of that cow changes from reproduction to supplying protein, this category would be the most difficult for the COP approach to estimate.

### Within-Sample Testing Results

Using the methods above within-sample comparison examined the accuracy of the three methods in determining animal values. This was achieved by forecasting monthly prices throughout 2016 and comparing the forecasted prices to the corresponding observed monthly market data. The year 2016 was chosen since it was the most recent complete year of data as of the time of this study. From a market perspective, 2016 was a year characterized by high beef and cattle market prices earlier in the year that declined throughout the year and recovered slightly in December. Typically, a cattle market cycle lasts 4–6 years, and 2016 fell mainly on the downside of that cycle. Pasture conditions were generally good in 2016, so no dramatic change in cow prices from stress-marketing was observed.

To achieve the best comparison possible, the Oklahoma observed market prices were transformed into a price per hundredweight ($/cwt) for cows sold and applied to eight representative females:

- 550 lb open replacement commercial heifer under 1 year of age of average quality
- 550 lb open replacement Angus heifer under 1 year of age of high quality
- 1,050 lb 2-year-old bred commercial replacement heifer of average quality
- 1,050 lb 2-year-old bred Angus replacement heifer of high quality
- 1,250 lb 5-year-old bred commercial brood cow of average quality
- 1,250 lb 5-year-old bred Angus brood cow of high quality
- 1,200 lb 10-year-old bred commercial brood cow of average quality
- 1,200 lb 10-year-old bred Angus brood cow of high quality

Actual values were calculated for each month in 2016 for these eight representative females by taking the price in $/cwt in that month and multiplying it by the weight of that female in cwt. These actuals were compared with the monthly price forecasts for the same eight described females using each method. Each method captures beef cattle characteristics differently. The hedonic pricing method is the only method that will allow for a price differential between the high quality Angus females and the average quality commercial females. The hedonic pricing and VECM methods allow variation across months, but the COP method gives an expected value across the entire year. These differences help capture the tradeoffs of estimating livestock values when different levels of data are available.

**Figure 3:**
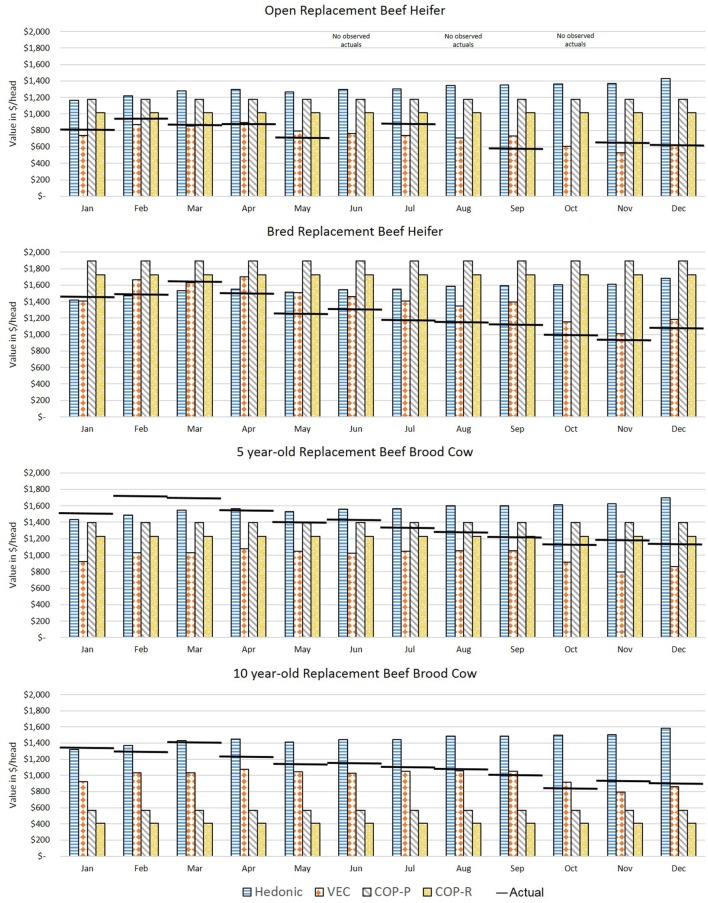
Comparison of forecasted and actual values from January to December 2016 for average quality replacement beef females. Forecasted values (vertical bars) versus actual values (horizontal lines) for each month of 2016 for average quality commercial beef females. Source: analytical results.

**Figure 4:**
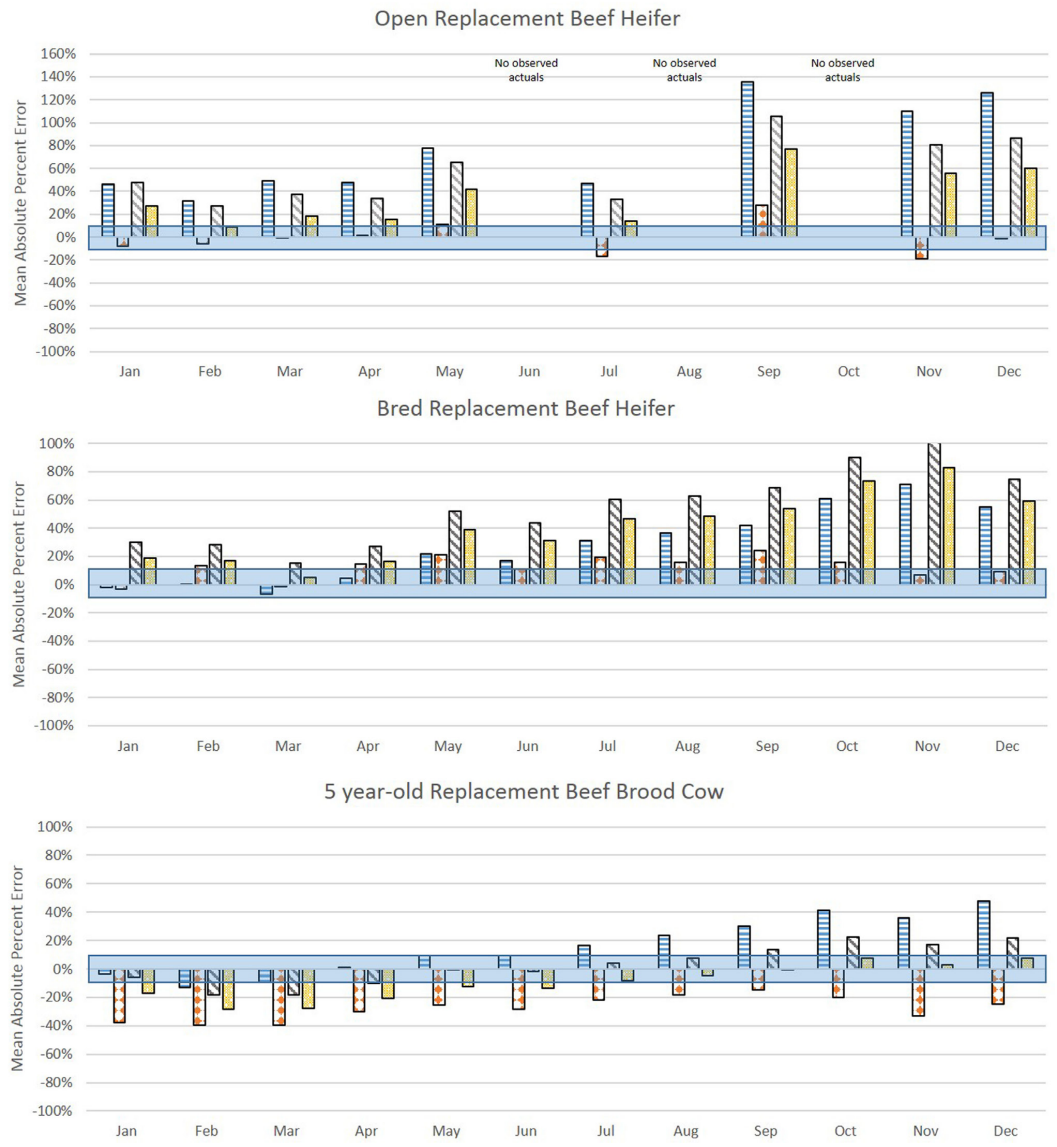

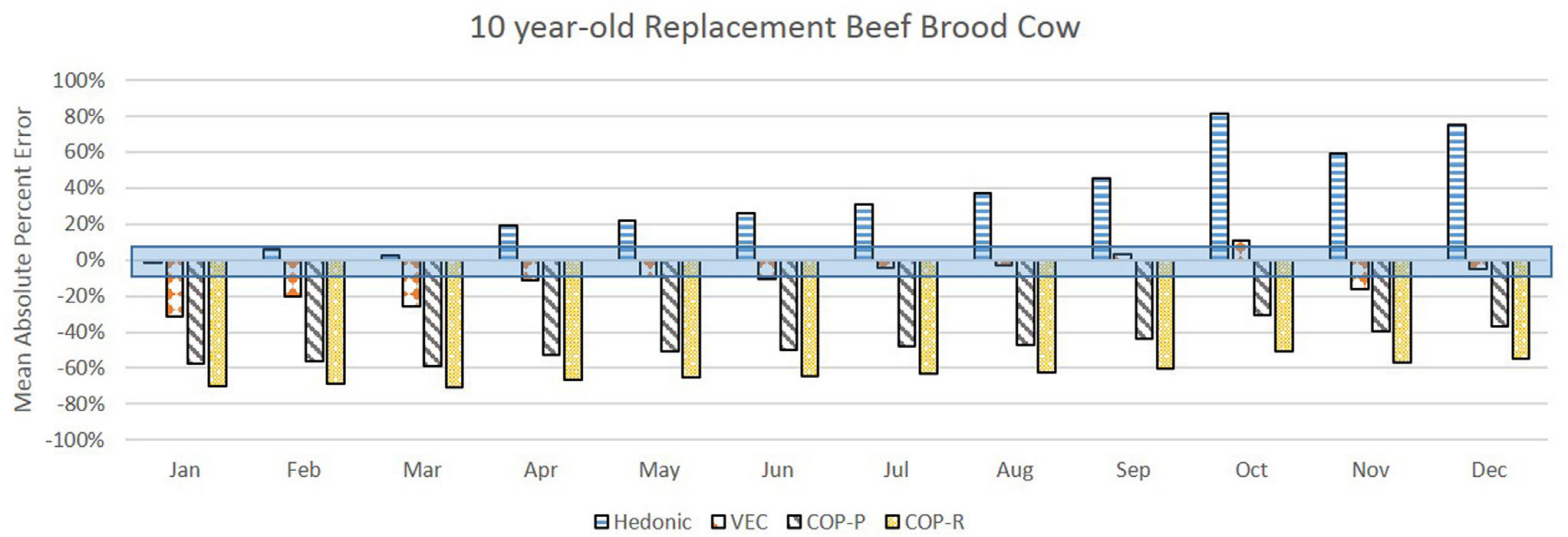
Mean absolute percent error (MAPE) of forecasted values compared with actual values from January to December 2016 for average quality replacement beef females. The shaded bar represents 10% above and 10% below a MAPE of 0%. Source: analytical results.

**Figure 5:**
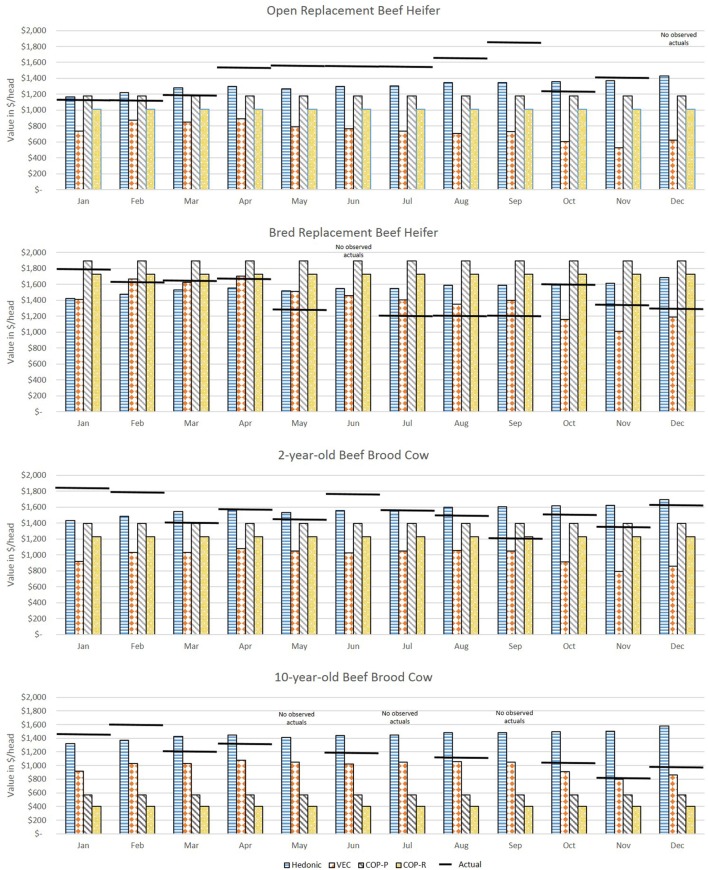
Comparison of forecasted and actual values from January to December 2016 for high quality Angus replacement females. Forecasted values (vertical bars) versus actual values (horizontal lines) for each month of 2016 for high quality Angus beef females. Source: analytical results.

**Figure 6:**
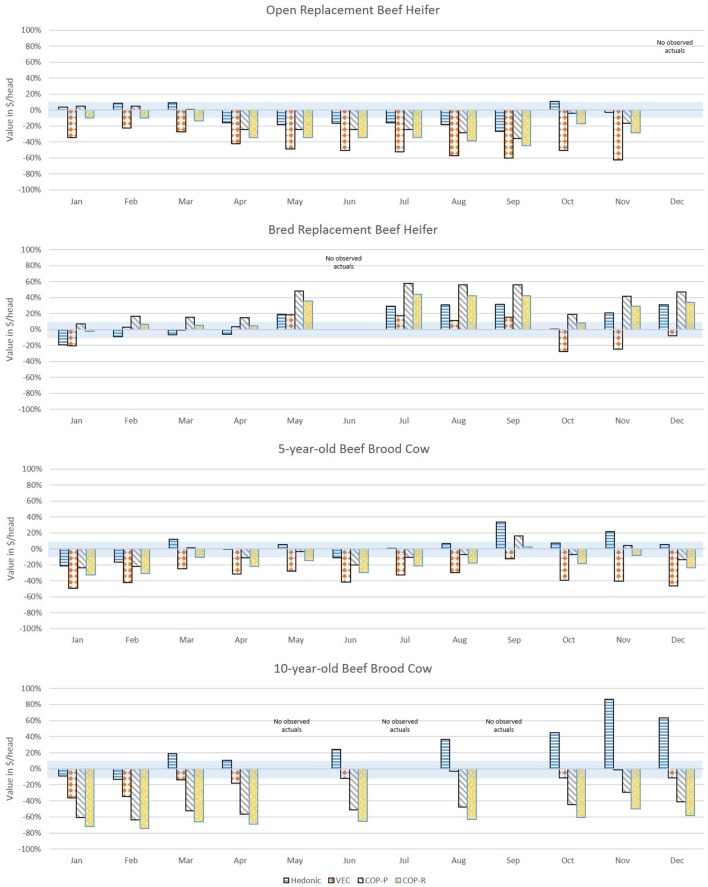
Mean absolute percent error (MAPE) of forecasted values compared with actual values from January to December 2016 for high quality Angus replacement females. The shaded bar represents 10% above and 10% below a MAPE of 0%. Source: analytical results.

Figure 3 shows the forecasted values (vertical bars) versus actual values (horizontal lines) for each month of 2016 for average quality commercial beef females. The MAPE for each comparison is in Figure 4 where the shaded box represents a 10% error above and below 0. Figures 5 and 6 show the forecasted versus actual prices for high quality Angus replacement females and the MAPE of each monthly comparison respectively.

For the heifers, most methods overvalued the representative heifers selected as compared with observed prices. The VECM performed well for the weaned heifer calves and replacement heifers, resulting in the lowest MAPE in 90% of the months for weaned heifers and 75% of the months for replacement heifers. The hedonic pricing method forecasted values that overestimated average quality females, more so for open heifer calves. Weaned calves were reported in the lot type pairs in the Oklahoma data. This diluted the reporting on the actual price of a weaned calf. Though the hedonic pricing method was the closest value for the high quality heifers, most of the time the methods overestimated or underestimated the high quality open heifers by more than 10%. This indicated that no one method may be best suited to valuing high quality heifers in a specialized market.

For 5-year-old brood cows, the MAPEs were overall lower as compared with younger or older beef females. This likely reflected how common it was in the data to have 5-year-old cows being sold for replacement. The COP method performed well when compared with the more data intensive econometric methods. This could be because the enterprise budget was reflective of the costs specific to Oklahoma. So, where the VECM in particular suffered from using data that were too aggregated, the COP offset that effect slightly. The COP method, where retained heifers and purchased heifers were combined, had the lowest MAPE in 75% of months for the 5-year-old average quality brood cow; hedonic pricing resulted in the lowest MAPE in the other 25% of the months. The lack of seasonal adjustments appeared to contribute to the error associated with COP, and particularly the effect of timing on breeding and retention decisions before winter when feeding was most intense. However, the hedonic pricing method tended to do much better when forecasting higher quality replacement cows as measured by the MAPE. The VECM results were often close to the results of the hedonic pricing model for bred, high quality replacement females.

The VECM chronically undervalued the 5-year-old replacement cows even after accounting for the influence of cull cows on the national price series. This likely reflected the averaging in the data series used, both across cow types (cull versus replacement) and across regions. The VECM forecast undervaluation was only exacerbated when examining high quality cows. The VECM did a better job of valuing cows later in the year as prices declined. This may reflect the relative strength of this estimation method in capturing price dynamics.

In the valuation of the 10-year-old brood cow that has a few productive years remaining, the COP method struggled to accurately forecast. Across methods, for a 10-year-old average quality brood cow the MAPE was under 10% in 8 of 12 months, and the VECM resulted in a MAPE under 10% in 5 of those 8 months. It may be easy to value a 10-year-old cow as a cull intuitively, but there may be times in the market, particularly during periods of herd expansion, when cows were retained longer or when other beef cattle producers were looking for “bargains” to get a few more calves on the ground. When the 10-year old brood cow was an Angus cow of higher quality, the ability to predict value was decreased. Across methods, the MAPE was under 10% in only 4 of 12 months. The VECM was often the closest estimate of the 10-year-old brood cows that were high quality, but always on the low side as compared with the actuals. For the hedonic pricing method, data may have played a role in the inaccuracy of results. There were fewer older brood cows sold on the market in Oklahoma compared with other types so the impact of individual characteristics may be heavily influenced by younger cows. Also, the hedonic pricing method did a poorer job of forecasting the mid-year market lows and overall declining trend through the latter part of the year. Instead the forecasted price remained fairly consistent through the 12 months varying by no more than $250.

There were months in which no observed data were available to compare to. These were months were it was less common to sell certain types of animals. As mentioned previously, most calving occurs in the spring and weaning occurs in the fall. So it was less likely that an observed market price for a weaned heifer was seen in the summer. An unexpected benefit of these results was the ability to see the ranges that prices might have fallen in during those months where no market data were observed for comparison.

## Discussion

An enhanced understanding of livestock valuation improves estimates of impacts due to production shocks and is an understandable metric of loss for decision makers in both the public and private sectors. The range of value differences across regions, time and animal types needs to be understood before determining whether comparable market data are appropriate for livestock valuation. Livestock valuation methods are often data intensive, requiring complete market data for a similar type of animal or region, extensive cost data, or survey implementation. The accuracy of livestock values estimation is inhibited by data limitations. The methods explored in this article may provide avenues to estimate value in a way that is accessible and consistent with economic theory. It is up to the individual researcher to determine the level of regional aggregation, time frame and tolerance for inaccuracy that is most appropriate for the question being asked.

This article applied three alternative methods to the valuation of bred replacement beef cows in Oklahoma and utilized data that were a proxy to varying types of market data limitations. The data used for the hedonic pricing method were limited by region, but rich in detailed animal characteristics. The vector error correction model data were limited in detail, but representative of a larger geographic space and accounted for upstream and downstream price impacts across time. The COP approach is limited in time variation but may be more accurate for a specific operation and does not require extensive time series data. Generally the direction and magnitude of the coefficients associated with factors impacting beef cow prices in the hedonic pricing method and vector error correction method align with what was seen in previous literature. Price forecasts for eight replacement beef cow or heifer types were created from each method, and compared in each month of 2016 to observed prices in Oklahoma. Results suggested that data intensive econometric approaches such as the hedonic pricing method or vector error correction model did approach the real values; however, where the hedonic pricing method overvalued replacement females (63% of MAPES were positive for average quality females and 60% of MAPES were positive for high quality females), the VECM method undervalued females (65% of MAPES were negative for average quality females and 84% of MAPES were negative for high quality females). The least data intensive method using COP data performed reasonably well for young and middle-aged cows, though the lack of seasonality impacted accuracy. It may be possible to refine these COP estimates by using seasonal indices to capture common market patterns on feed input costs and cattle prices received.

Within-sample forecasting results suggested that all three methods more frequently overvalued monthly values for heifers in 2016 and all three methods did a poorer job of valuing 10-year-old brood cows that may have some remaining productive life. Examining each method individually across all eight beef female types, for average quality beef females the VECM forecast resulted in a MAPE under 10% for 33% of forecasted months, followed by hedonic pricing at 24% of the forecasted months and COP at 14% of the forecasted months. For high quality females, the hedonic pricing method worked best producing a MAPE under 10% in 36% of the forecasted months followed by the COP method at 21% of months and the VECM at 14% of the forecasted months.

There is a tradeoff between data intensity and forecast accuracy, and some general conclusions were made from this analysis. First, given that certain methods performed better than others in this application, researchers tasked with livestock valuation may need to utilize different valuation methods—or even multiple valuation methods—depending on the type of livestock affected and the types of market transactions data available. Second, the context of the data mattered for the accuracy of the forecasts. Consider the VECM results which ranged from a MAPE of less than 1% up to a MAPE of 60% when compared with observed Oklahoma bred replacement beef cow prices. However, performing the same within-sample forecast comparison against the observed values of the aggregated national cow prices the VECM was based on, the MAPE was less than 1% on average and never exceeded 10% error in any given month. Thus, the majority of the error was in the application of the results to the Oklahoma bred cow dataset, and particularly the high quality bred cow data set. Third, in several months the MAPES were quite large and well above what would be considered a reasonable tolerance for error. This indicated that even in a situation where market data were available that would appear to be sufficient for quantitative analysis, researchers should be aware of the potential impacts of data constraints. It is possible that data could have been cut further for the hedonic pricing method and a different model could be estimated for each of the eight cow types. The aggregation in the national data used for the VECM would not allow for that approach to break down types down further to test for increased estimate accuracy.

Focusing this analysis in Oklahoma had benefits. It was possible to quantitatively measure forecast inaccuracy because a bred replacement beef cow data set was available, and Oklahoma has regional importance for cattle production. However, there are few other regions of the United States that have these same data availability and it is difficult to say whether conclusions could be extended to other geographic regions. Further analysis would be needed to determine the extent to which these conclusions hold in different phases of the marketing cycle—for example, when beef cow herd inventories are expanding resulting in an upward trend in prices.

Other methods such as stated preference or contingent valuation could be examined to determine whether forecast accuracy is improved. For example, stated preference elicitation may be useful, particularly if characteristics affecting the value for an animal were less tangible, such as livestock serving as a status symbol, providing draft power, or livestock production as a hobby. Another, more complex approach of price differential modeling could be used to estimate the cattle prices in the United States as well. Applications of price differential modeling suggested that the approach provided a useful supplement to market data in the short-run, but does not provide complete data to cattle owners for decision making in the long-run. In addition, there are other approaches to estimate the profit applied back to each cow by manipulating known market data. Such applications were beyond the scope of this study. Alternative methods to valuing losses that use different types and intensities of data are available and can be employed within a degree of error when doing livestock valuation. It is hoped this study encourages continued innovation of ways to utilize constrained market data for livestock valuation, because market data are likely to be an increasingly scarce resource into the future.

## Author Contributions

AH was the project leader, estimated the VECM, and lead manuscript development. JT estimated the hedonic pricing model and contributed to manuscript development. CH and KJ estimated the cost-of-production method and contributed to manuscript development. All the authors met the contribution requirements for authorship per the author guidelines.

## Conflict of Interest Statement

The authors declare that the research was conducted in the absence of any commercial or financial relationships that could be construed as a potential conflict of interest.

## Footnotes

^1^Market thinness, in this study, can refer to too few markets, too few cattle being sold in markets, or too infrequent sales in an area.
